# My virtual escape from patient life: a feasibility study on the experiences and benefits of individualized virtual reality for inpatients in palliative cancer care

**DOI:** 10.1186/s12904-024-01577-2

**Published:** 2024-10-23

**Authors:** Christina Gerlach, Laura Haas, Anja Greinacher, Jonah Lantelme, Melanie Guenther, Julia Thiesbonenkamp-Maag, Bernd Alt-Epping, Cornelia Wrzus

**Affiliations:** 1 Department of Palliative Medicine, Heidelberg University Hospital, Im Neuenheimer Feld 305, 69120 Heidelberg, Germany; 2 Clinical Psychology, Interaction- and Psychotherapy Research, Faculty of Social Sciences, University of Mannheim, L54, 68161, Mannheim, Germany; 3 Psychological Institute and Network Aging Research, Ruprecht Karls University of Heidelberg, Bergheimer Str. 20, 69115 Heidelberg, Germany

**Keywords:** Virtual reality, Innovation, Palliative care, Oncology, Hematology, Personalized therapy, PROM, Well-being

## Abstract

**Background:**

Cancer patients benefit from Virtual Reality (VR) in burdensome situations, but evidence is scarce for palliative situations. Based on earlier work in palliative care, individualized VR interventions like seeing the patient’s home may address a patient’s wish to be at home and thus have a greater effect compared to standard VR content. Yet, some patients and relatives may be concerned about their privacy. Also, patient stakeholders raised concerns about triggering depressed mood or homesickness.

**Aim:**

To test the feasibility and safety of individualized vs. standard 360°video VR interventions in palliative cancer inpatients.

**Methods:**

Prospective observational study with patient-reported outcome measurement using validated instruments of well-being (MDBF), symptoms and psychosocial burden (IPOS), cybersickness (SSQ), presence experience (SPES), subjective benefit (2 items), content analysis of interviews, and field notes. Individualized VR content was recorded with action camcorder-technology to protect the patients’ privacy.

**Results:**

Seventeen patients participated, median age 65 years (range 20–82), 9 women (53%), 8 single or widowed (47%), 4 childless (23.5%), 4 academics (23.5%), with a median length of stay of 9 days (1–75) in the hematology (10), palliative care (3), or radiotherapy (2) unit of a German university hospital. Eight patients (53.3%) chose their own home environments or family for individualized VR-content. All participants enjoyed the intervention. Compared to standard VR content the individualized VR tended to have a stronger effect on well-being and emotional touch. It was not inferior in terms of psychosocial burden and cybersickness. No subjective and relevant side effects occurred. The patients well tolerated the assessments. However, most patients demanded a lighter headset and a desire for more interactivity.

**Conclusions:**

Individualization of VR content shows potential for enhancement of immersion, which improves the VR experience and does not harm in terms of depressed mood or worsening of symptoms. The patients’ and family desire for privacy is feasible with the support of family members who recorded the individualized videos, which is easily manageable today. We suggest a pragmatic randomized clinical trial to compare the effects of individualized vs. standard VR-content.

**Trial registration:**

Registered at German Clinical Trials Register (Deutsches Register Klinischer Studien; DRKS); registration number: DRKS00032172; registration date: 11/07/2023.

**Supplementary Information:**

The online version contains supplementary material available at 10.1186/s12904-024-01577-2.

## Introduction

Given the growing need for palliative care, the development of innovative interventions for effective control of symptoms, psychosocial and spiritual burden is becoming increasingly important. Demographic change and improved survival times even in incurable situations lead to an increase in palliative cancer patients requiring inpatient care although they may prefer being at home. How would patients feel if they could virtually escape from hospital to their individual comfort area? Virtual Reality (VR) gives patients the technical opportunity to move around in environments that resemble objects and events in the real world, to “immerse” themselves in the simulated environment and to create a feeling of “presence” in the virtual world [1]. The basic principle of VR interventions is to distract the patient’s senses from real stimuli and replace them with simulated stimuli. Cancer patients benefit from VR in burdensome situations [2], but evidence is scarce for palliative cancer patients [3, 4]. VR interventions potentially improve their well-being and reduce the perception of symptoms [5], but the impact of the shown VR content it is still unclear. Here, the possibility of personalized content is considered particularly promising [6–9]. In the clinical context of VR ‘personalization’ means at the patients’ choice from a selection of standard, mostly nature sceneries [6, 7]. In our study we take an even more rigorous approach to person-centered personalization and apply ‘individualized’ VR, i.e. content produced for the individual patient of his/her choice.

This study is part of a larger project to investigate whether individualized VR videos have additional benefits over standard VR on symptom relief, well-being, treatment satisfaction, and adherence in patients under palliative cancer care [10]. First, we assessed patients’ and relatives expected benefits and concerns about individualized VR content [11, 12]. Although, individualized images, e.g. the patient’s home environment, may have a greater emotional impact, some patients and relatives were concerned about their privacy. A member of the patient advisory board had concerns about triggering depressed mood or homesickness. Thus, we conducted a study to test the clinical feasibility of an individualized VR intervention in palliative cancer inpatients. We tested standard vs. individualized 360° VR videos, both at the patients’ choice, in terms of possible effects and side effects from the patients’ perspective regarding well-being, symptoms and psychosocial burden, cybersickness, presence experience, subjective and noticeable benefit. Only if the intervention is well tolerated and safe for the patients an RCT (randomized controlled trial) would be conducted.

## Methods

In a previous part of the project, we interviewed patients, relatives and the patient advisory board about their wishes and concerns regarding the intervention. The intervention study described here is followed by a Patient and Public Involvement (PPI) focus group to discuss the results and further implementation [10, 12].

### Study design and setting

This study is an interventional prospective clinical cohort study conducted at the University Hospital of Heidelberg. Terminally ill patients, hospitalized on the hematology, radiology, or palliative care wards, received a VR intervention consisting of an individualized and a standard VR video and were followed up for two weeks between December 2023-April 2024. We pursued an analytical approach that integrates quantitative and qualitative methods.

### Participants

Inclusion criteria were ≥ 18 years of age, a diagnosis of an incurable cancer or uncertain prognosis of a severe hematological malignancy (HM), and ability to give informed consent. We excluded patients who, in the opinion of the attending physician, were in poor general health or too distressed to participate in any study, had cognitive or communication deficits, or had a life expectancy of only a few days.

### Intervention

All participants experienced two VR contents. Based on our previous study exploring VR-needs of patients and relatives [11, 12], in the individualized intervention condition, participants were asked about their preference for individually recorded video content. The patients' relatives were then asked and enabled to record videos of the patients’ home and/or family with a 360° action camcorder (GoPro MAX, GoPro, Inc., San Mateo, CA, USA). If no relative was available, a research assistant recorded the video. In addition, we created especially for the project a VR-walk through Heidelberg, on the assumption that this could be individually meaningful for some patients. The videos were edited using Adobe Premier Pro (Adobe Systems Software Ireland Limited, Dublin, Ireland) and transferred to the HMD device (head mounted display).

In the standard condition, participants selected one of eight standard 360° videos showing scenes of, for example, a forest, a beach, Paris, accessed via the YouTube VR video platform (Google Ireland Limited, Barrow Street, Dublin, Ireland). To ensure comparability all videos ranged in the same length (4:30–7:00 min).

All VR videos were presented on the tried-and-tested Meta Quest 2 virtual reality HMD device (Meta Platforms, Inc., Menlo Park, CA, USA) [13–16]. The headset of 503 mg weight is a wireless standalone device that requires no external tracking system and has built-in speakers. Adjustable straps (Meta Quest 2 Elite Strap) were used to make the headset easy and comfortable to wear. The devices were disinfected after interventions and disposable headset inserts were used.

After the end of the study, we provided the patients with their individual video files on a USB device, which allowed them to watch the videos on a regular PC, as well as the sources of the standard videos. 

### Data collection

The primary outcome measure was patients' self-reported well-being using a six-items short form of the Multidimensional Well-Being Questionnaire (MDBF) [17]. The MDBF measures the current emotional state on three bipolar dimensions (good-bad mood, alertness-tiredness, and calmness-restlessness) on a five-point Likert-scale, with the endpoints characterized by opposing adjectives (e.g., very tired—very alert). High scores indicate the positive pole with single, and total scales from 2–10, and 6–30.

To assess physical symptoms, emotional concerns as well as communication and practical problems, the patients completed the Integrated Palliative Care Outcome Scale (IPOS) (https://pos-pal.org/). It is a self-reported 17-item multidimensional scale designed to identify the main concerns of patients in palliative care. Each item is answered on a five-point Likert-scale with total IPOS-score from 0–68, physical symptoms score 0–40, emotional symptoms score 0–16, and the communication/practical problems score 0–12, with higher scores indicating more subjective burden [18].

We assessed cybersickness after the VR intervention using the Simulator Sickness Questionnaire SSQ [19]. The questionnaire asks about seven symptoms such as nausea, dizziness, headaches, using a 0 “not at all” to 6 “very strongly” scale. Total scores ranged from 0–42, with higher scores indicating more pronounced cybersickness. The experience of presence in VR was measured using the short form (8 items) of the Spatial Presence Experience Scale SPES [20]. Items such as “I felt like I was actually there in the environment of the presentation” were rated on a seven-point Likert-scale with total SPES scores from 8–56. Higher scores indicated a greater experience of actually being within the VR environment.

Also, we assessed the subjective benefit using two self-developed items: "It helped me to watch the video" and "I think the use of such videos makes sense", which were answered on a 7-point scale from "not at all" to "very much". After the intervention, we explored the patient’s experience conducting a short, structured interview about the video content, the use of VR, the comfort of the headsets, and suggestions for improvement.

**Fig. 1:**
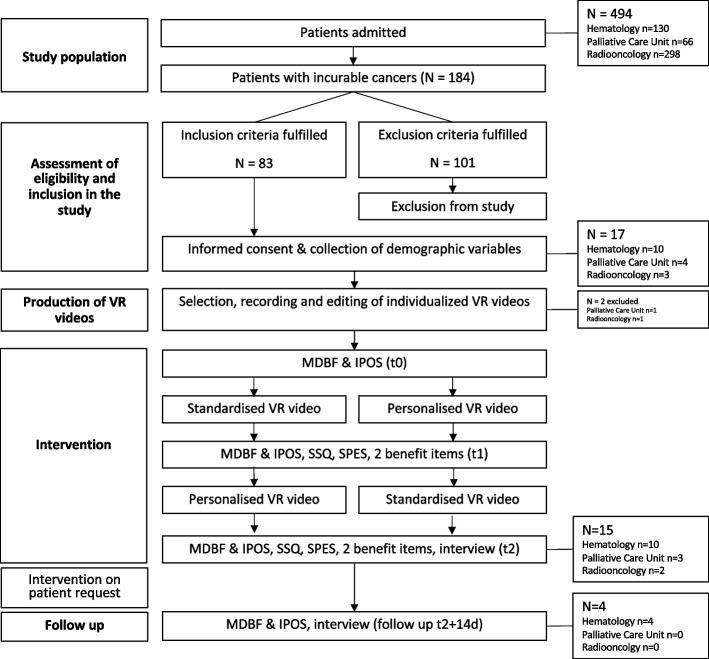
Flow chart of participation. t0 = baseline, t1 = after the first VR, t2 = after the intervention. IPOS, Integrated Palliative Outcome Scale; SSQ, Simulator Sickness Questionnaire; SPES, Spatial Presence Experience Scale

The research assistants took structured field notes after all measurement time points. Assessments took place before (t0) and after (t1) the first video, after the second video (t2), and at two weeks thereafter (follow up) with MDBF and IPOS at all measurement time points, SSQ and SPES at t1 and t2, and the structured interview at t2 (Fig. 1).

Depending on their individual preferences and physical conditions, patients were either lying in bed or sitting in a chair during their VR sessions. Participants physically unable to complete the questionnaires were supported by the study staff. The entire procedure took approximately 30–45 min.

Physician support was available at all times of the intervention in case of any symptoms or other burden reported by the patients or the trained study staff.

### Data analysis

We performed a descriptive analysis with the total data set reflecting the intention-to-treat principle using Jamovi Software (The jamovi project, Sydney, Australia, Version 2.3 retrieved from https://www.jamovi.org). We summarized continuous variables by the frequency distributions, mean (M), median (Md) and standard deviation (SD). Categorical variables are presented as absolute and relative frequencies*.*

CG (palliative care specialist), JL (research assistant), and JTB (sociologist) performed the content analysis of the post-intervention interviews based on subtle realism [21, 22], and compared the results with observations from the fieldnotes.

## Results

### Participants

Of 494 patients admitted during the study period 184 suffered from incurable cancers, 83 were eligible, and 17 patients gave informed consent to participate (Fig. 1). The mean age was 65.5 (SD ± 13.0, range 20–82 years), 9 were women (52.9%). The number of days spent as an inpatient ranged from 1 to 75 days (mean 23.4, SD ± 27.1). Two patients dropped out of the intervention because the relatives did not support their preferred video recordings at home (Fig. 1). These two patients were offered the standard VR, but they did not use it. Demographic data are shown in Table 1.

**Table 1:** Demographic data of the entire sample

Demographic data		
	*n*=17	
	value	%
Age		
M (SD)	65.5 (13.0)	
Median	65	
Min	20	
Max	82	
Sex		
women	9	52.9%
men	8	47.1%
Level of education		
high school with vocational focus (preparing for further vocational training; similar to finishing schools after GCSE's; apprenticeship)	11	64.6%
high school with academic focus (preparing for university; similar to A-levels, grammar school upper secondary level)	1	5.9%
college or university	4	23.5%
other	1	5.9%
Gross income in € per month		
<1500	4	23.5%
<2500	2	11.8%
<3500	4	23.5%
>4000	1	5.9%
Not specified	6	35.3%
Marital status		
single/ currently no partnership	7	41,10%
married/ currently in a stable partnership	9	52.9%
widowed	1	5.9%
Number of children		
no	4	23.5%
1	5	29.4%
2	6	35.3%
>2	2	11.8%
Duration of hospitalization		
M (SD)	23.4 (27.1)	
Median	9	
Min	1	
Max	75	

*M* mean value, *SD* standard deviation, *Min* minimum, *Max* Maximum

### Patient choice of virtual reality content

Ten patients (66.6%) had no prior VR experience. For the individualized VR content, three patients (20%) chose to record their home, five (33.3%) their loved ones and four (26.7%) indicated other meaningful locations: their hometown (1), a specific mountain racetrack (1), a specific farm (1), and local forest trails (1). Three patients (20%) decided to watch the Heidelberg video. For standard VR, 12 patients (80%) chose a nature shot, and three (20%) preferred a city scene.

### Effects of virtual reality on physical and psychosocial burden

**Fig. 2:**
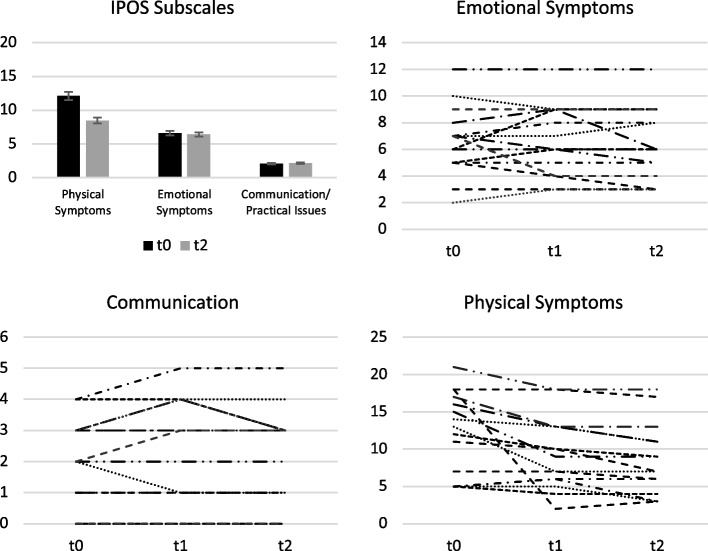
Physical and psychosocial burden as reflected by the IPOS subscales. Mean values ± SE (bar chart) and individual changes (spaghetti diagrams) across all measurement time points. Lines of the spaghetti diagram represent the scores of the individual patients. SE = standard error. t0 = baseline, t1 = after the first VR, t2 = after the intervention. Higher scores are associated with a greater symptom burden. n = 14/6/2 patients (pts) improved, *n* = 0/4/10 pts stayed the same, and n = 1/5/3 pts deteriorated on the symptom/emotional/practical-communication subscale after the VR intervention

Patient symptom burden did not differ between the individualized and the standard VR intervention. The mean IPOS total score was 17.7 (SD ± 6.44) after individualized VR and 17.5 (SD ± 6.30) after standard VR. Patients' symptom burden tended to decrease with each measurement time point from 20.8 (SD ± 7.15) to 17.0 (SD ± 6.27) before and after the VR intervention (Fig. 2). This trend appears to be more pronounced regarding the physical symptom subscale of the IPOS (from 12.1, SD ± 5.54 to 8.47, SD ± 4.81) than the emotional symptom subscale (from 6.60, SD ± 2.56 to 6.40, SD ± 2.69) and communication (from 2.07, SD ± 1.39 to 2.13, SD ± 1.55) with intra-individual variation (Fig. 2).

### Effects of virtual reality on well-being

**Fig. 3:**
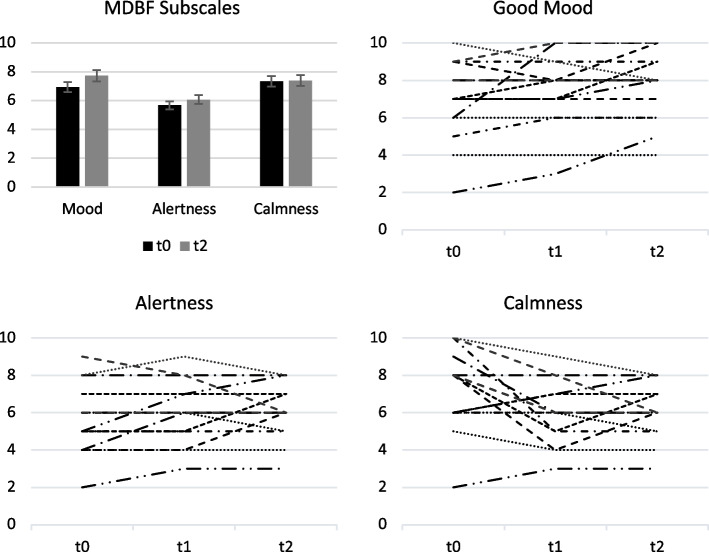
Well-being as reflected by the MDBF subscales. Mean values ± SE (bar chart) and individual changes (spaghetti diagrams) across all measurement time points. Lines of the spaghetti diagram represent the scores of the individual patients. SE = standard error, t0 = baseline, t1 = after the first VR, t2 = after the intervention. Higher scores are associated with the positive pole of the scales bad-good mood, tiredness-alertness, restlessness-calmness. In *n* = 8/5/3 of the patients (pts) the scores improved, in *n* = 6/5/2 pts stayed the same, and in *n* = 1/5/10 pts the scores moved in the direction of the opposite pole of the mood/alertness/calmness subscale after the VR

**Fig. 4:**
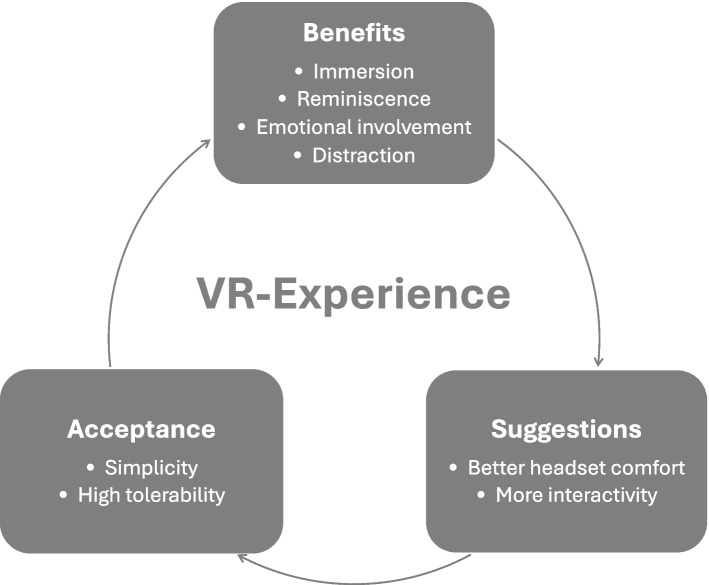
Thematic model of the Virtual Reality experience. The model based on the content analysis of the post-interventional interviews

The primary outcome well-being did not differ between the individualized and the standard VR-intervention. The mean MDBF-total score after the VR-presentation was 21.1 in both cases (SD ± 4.79 for individualized VR; ± 4.61 for standard VR). Also, the subscale results did not differ. The patients’ well-being tended to increase with each measurement time point from 19.9 (SD ± 5.54) to 21.2 (SD ± 4.25) before and after the VR-intervention (Fig. 3). The trend seems to be mainly due to the mood-subscale of the MDBF (from 6.93 ± 2.12 to 7.73 ± 1.83), rather than the alertness subscale (from 5.67, SD ± 1.84 to 6.07, SD ± 1.44), and calmness-subscale (from 7.33, SD ± 2.19 to 7.40, SD ± 1.64) (Supplement 1). The intra-individual courses varied, with the greatest heterogeneity in the calmness subscale. These observations correlate with the results from the qualitative analysis of the post-interventional interviews (Fig. 4).

### Follow-up assessment after two weeks

Four participants used the opportunity to watch their individualized VR for two weeks with 1–3 repetitions. In the follow-up survey, the subjects' mean overall MDBF score remained stable compared to t2 (20.5, SD ± 4.80). However, the mean overall IPOS score had deteriorated to 35.3 ± 9.60 at follow-up compared to 21.3 ± 9.0 after the intervention.

### “It was the biggest gift”—post-interventional interviews

Most patients did not attribute the benefit of the VR to a state of calmness, but rather to feeling emotionally moved in a positive way, although the feelings fluctuated between joy and homesickness.

“So I thought that was good. I saw my home. (sobs). The family (……) The garden. The garden. [break]: Just the things, I'm missing. I think that’s it.” (R04w).

„I thought the video with the relatives was very, very good. It touched me deeply emotionally. All my family members, my little sister, my relatives were there too – just emotional. It put me into a good mood.“ (A02m_ T1).

In this context, some patients welcomed the option to escape from their patient situation, one patient reported to be explicitly distracted from pain.

“It took me out of it a bit when I was in such enormous pain and made me forget a few other things, too. And I thought that was amazing and I thought that was very good. I would recommend it to everyone.“ (A08w_ T1).

„What I liked about the private video was that you can just be, where you like to be.“ (A10w_ T1).

The content analysis of the post-interventional interviews suggests that the experience of presence and memories to which the virtual reality is linked make up the patient perceived benefit (Fig. 4).

“It was very realistic, especially in the video where I was at home. I thought, where have I ended up? I really thought I was at home in the living room. I have to say it was very realistic, simply in terms of the vibes.“ (A02m).

Overall participants rated their presence experience (SPES) very high for the individualized videos with 5.97 (SD ± 1.40), and 5.42 (SD ± 1.46) for the standardized videos (Table 2). The immersion was also observed by the study staff with patients’ expressions of joy and interest during most of the interventions, as well as numerous head and body movements.

**Table 2:** Spatial Presence Experience Scale (SPES) data

		Individualized Video	Standardized Video
Overall Score			
	*M*	5.97	5.42
	*Md*	6.38	5.88
	*SD*	1.40	1.46
Self Location			
	*M*	6.02	5.40
	*Md*	6.25	6.00
	*SD*	1.39	1.47
Possible Actions			
	*M*	5.93	5.43
	*Md*	6.25	5.50
	*SD*	1.42	1.53

*M* = mean value. *Md* = median. *SD* = standard deviation

Scale ranging from 1–7. Higher values indicate greater presence experience

Besides individual meaningful memories patients mentioned technical characteristics that supported the immersion like the 360° perspective, vivid colors, natural noises and music.

“The realism. It is really impressive that you can put yourself out there in the world like that, above all, 360 degrees ALL AROUND. So you can look in all directions and that's amazing.“ (A03m).

„Related to the first [individualized VR], it gives you a piece of home. (..) And with the second one [standard VR], also memories were brought back. And that was… I chose it so I could see the sea because I love the sea. And I really liked the sound of the waves and so on. And the colors, everything so realistic. And is, um, did not only evoked longing, but also a bit of hope [weeps]“ (A08w).

Patients who participated in the follow-up preferred their individualized VR content. However, one patient explained different impacts of the two VR contents: his standard VR, a generic beach scenario that reminded him of his favorite holiday destination, calmed him down, the individualized conveyed motivation and a deep joy. Another patient even was afraid to be bored of repeated standard VR content.

“It wasn't boring [the standard VR], you could watch it, but it was a bit off compared to the first one [the individualized VR].” V01w.

### Headset comfort

A clear suggestion of the patients was to improve the HMD in terms of weight and accumulated heat. Most patients needed support to install the HMD, in particular patients with glasses. Further, they wished for more interaction in terms of ease of use e.g. to fast forward, but also to interact with the VR.

### Acceptance

On average, patients answered the statement "It helped me to watch the video" with 5.47 (SD ± 1.81) and "I think the use of such videos makes sense" with 6.10 (SD ± 1.42), indicating that they find the VR intervention beneficial both for themselves and in general. Some patients were grateful for the VR-intervention and encouraged the study staff to carry on.

“I like what you're doing, yes. And, um, it's very gratifying, yes, that you're offering something like that and all I can really say is, keep it up.“ (A09w).

We observed no serious side effects (Supplement 2) and no participant requested to stop the VR intervention.

## Discussion

We tested the feasibility and safety of a VR-intervention with individualized content in 15 palliative cancer and hemato-oncology inpatients in three departments of a German university hospital. Other than assumed from previous exploration regarding expected benefits and concerns of patients [11, 12], most participants desired and enjoyed individualized VR content with home environment. Symptoms and psychosocial burden did not worsen during use of the individualized VR, and the well-being even tended to be improved compared to standard VR. Moreover, in patients who used it repeatedly during two weeks, the well-being scores remained stable although the patients’ symptoms deteriorated. These results speak against a pronounced risk of inducing homesickness through individualized VR.

Still, other than previous studies that identified the potential of personalized and individually meaningful VR applications [5, 7–9, 23], we did not find substantial quantitative advantages over standard VR. This difference may be attributed to the design of the studies and the focus on symptom control [5, 7, 8, 23]. Limitations from our design may be the assumption that the Heidelberg VR-walk was individually meaningful rather than another standard, diluting the contrast. In this context, spillover-effects may also play a role because the patients watched the two VR-videos one after the other, thus, stimuli from the first VR-experience may impact the outcome after the second assessment. Nevertheless, our post-interventional interviews with the participants revealed a subjective benefit. Patients reported being emotionally touched, in particular when VR-content was associated with memories, recognition and reminiscence. This happened in our study with standard VR-content reminiscent of a trip to Paris, which Moon et al. [24] and others would define as ‘personalized’ content. Nevertheless, this memory-effect was frequently observed with individualized VR-content of the home environment and family, and reminiscence was identified to be the effective element to support immersion in VR-interventions even for patients with dementia [25–27]. Yet, memories may not always trigger increases in well-being. The participants of our study rated VR as beneficial, but individualized VR not as superior. The interviews contained echoes of homesickness and deep emotions. But even though some patients cried with emotion, they rated the VR positively and even expressed a desire for opportunities for more interaction. Researchers from Japan made similar observations in four palliative care inpatients, who immersed in a VR-supported phone-call with their relatives during the pandemic [14]. Although the VR-intervention succeeded in improving the patients’ feeling of normalcy and closeness to the family, the patients also expressed loneliness conveyed by the awareness of being separated from the family. This kind of experience made some patients in our study feel motivated and hopeful. Seiler et al. [15] described this distraction from patient reality. Thus, individualized VR-content may have the potential to bridge with virtual escapes from the patient reality to previous normalcy and to support coping as ‘double awareness’ [28, 29]. The term ‘double awareness’ describes ‘a person’s capacity to be engaged in the world while preparing for impending death’ [28]. VR in the hospital room could have an impact on this fine balance between the awareness of the end of life and hope in the present moment that supports the serious ill to cope with their situation.

In summary, the VR was well accepted and tolerated in this vulnerable patient group. Maintenance of well-being and symptom control was similar for standard and individualized VR videos, speaking to distraction and elicitation of positive mood as common mechanisms. Potentially, individualized VR videos could be beneficial for repeated use as subjective well-being of patients was stable despite physical declines, yet this observation needs to be substantiated in a larger sample. The need for privacy was met in nearly all cases with enabling the relatives to collect the virtual home recordings using an action camcorder. However, two patients dropped out, because their relatives did not consent in the home recordings.

### Limitations and strength

The needs of the relatives with objections had to be considered more pronouncedly. Another important point was the omission to systematically assess the migration background of the participants to better describe the diverse needs of patients in palliative care. From field notes and the interviews was apparent that migrant patients were well represented. Nevertheless, this study adds to the current knowledge, because it is the first systematic clinical testing of individualized virtual reality. Following the concerns of patients, relatives, and the patient advisory board, we aimed to test the feasibility and safety of the VR-intervention to prepare a larger study. We used mixed methods to approach both, the size and the way of possible effects. Another strength is the good representation of palliative hematology patients whose access to palliative care is more restricted compared to other oncological patients [10, 30, 31].

## Conclusion

### Research implications

This study aimed to test the feasibility of both, the approach and the investigation of individualized VR-intervention. To prove the added value, significant effects and the role of individualization, a powered comparative study needs to be initiated [32–34]. Besides feasibility of the outcome measurement instruments, we identified 0.5 SD of the MDBF well-being scale to be a useful minimally clinically important difference as a basis for calculation of the sample size. Moreover, quantitative methods indicating the effect size should be complemented by a qualitative approach to further explore characteristics of the effects as the latter proofed to be invaluable to this project. Also, further aspects of the intervention should be explored in future studies, e.g. the impact that the VR intervention may have on relatives. The next step of the project is to involve again the patient advisory board as well as healthcare professionals from the participating departments to determine gaps of research and the clinical implementation strategy.

### Clinical implications

Potentially negative effects from individualized VR-content, e.g. homesickness, need to be considered. The best prevention is to ask the patients, but also relatives, about their needs and surrounding circumstances to support self-selection for appropriate individualized VR-interventions. Thus, individualized VR-interventions could complement medical care and support quality-of-life at the end-of-life.

## Supplementary Information

Supplementary Material 1.

Supplementary Material 2.

## Data Availability

The datasets generated during the current study are not publicly available due to sensitivity but are available from the corresponding author on reasonable request.

## Abbreviations

HMD: Head Mounted Display
IPOS: Integrated Palliative Outcome Scale
MDBF: Multidimensional Well-Being Questionnaire
PPI: Patient and Public Involvement
PROM: Patient Reported Outcome Measurement
SSQ: Simulator Sickness Questionnaire
SPES: Spatial Presence Experience Scale
VR: Virtual Reality
