# Supplementary material for: My virtual escape from patient life: a feasibility study on the experiences and benefits of individualized virtual reality for inpatients in palliative cancer care
Source: BMC Palliat Care. 2024 Oct 23;23:247. doi: 10.1186/s12904-024-01577-2 (PMC11515567; doi:10.1186/s12904-024-01577-2)
Supplement: Supplementary file 1 — Supplementary Material 1. [file 12904_2024_1577_MOESM1_ESM.docx]

| **Supplement 1***.* Multidimensional Well-Being Questionnaire (MDBF).  Mean, median, and standard deviation of the MDBF by subscales and time point. | | | | | |
| --- | --- | --- | --- | --- | --- |
|  |  | Mood | Altertness | Calmness | Overallscore |
| *M* |  |  |  |  |  |
|  | t0 | 6.93 | 5.67 | 7.33 | 19.90 |
|  | t1 | 7.33 | 6.00 | 7.67 | 21.00 |
|  | t2 | 7.73 | 6.07 | 7.40 | 21.20 |
| *Md* |  |  |  |  |  |
|  | t0 | 7.00 | 6.00 | 8.00 | 20.00 |
|  | t1 | 8.00 | 6.00 | 8.00 | 22.00 |
|  | t2 | 8.00 | 6.00 | 8.00 | 23.00 |
| *SD* | |  |  |  |  |
|  | t0 | 2.12 | 1.84 | 2.19 | 5.54 |
|  | t1 | 1.99 | 1.65 | 2.09 | 5.11 |
|  | t2 | 1.83 | 1.44 | 1.64 | 4.25 |
| *Notes*. *M* = mean value. *Md* = median. *SD* = standard deviation. t0 = before the intervention, t1 = after the first VR, t2= after the intervention. Higher scores are associated with the positive pole of the scales bad-good mood, tiredness-alertness, restlessness-calmness. | | | | | |
