# Supplementary material for: My virtual escape from patient life: a feasibility study on the experiences and benefits of individualized virtual reality for inpatients in palliative cancer care
Source: BMC Palliat Care. 2024 Oct 23;23:247. doi: 10.1186/s12904-024-01577-2 (PMC11515567; doi:10.1186/s12904-024-01577-2)
Supplement: Supplementary file 2 — Supplementary Material 2. [file 12904_2024_1577_MOESM2_ESM.docx]

| Supplement 2. | |  |  |  |  |
| --- | --- | --- | --- | --- | --- |
| Simulator Sickness Questionnaire (SSQ) data | | | | |  |
|  |  | | Individualized Video | Standardized Video |  |
| Overall Score | | |  |  |  |
|  | *M* | | 5.73 | 5.07 |  |
|  | *Md* | | 2.00 | 3.00 |  |
|  | *SD* | | 6.84 | 6.26 |  |
| General feeling of discomfort | | | |  |  |
|  | *M* | | 1.40 | 1.30 |  |
|  | *Md* | | 1.00 | 1.00 |  |
|  | *SD* | | 1.76 | 1.54 |  |
| Headache |  | |  |  |  |
|  | *M* | | .67 | .67 |  |
|  | *Md* | | 0 | 0 |  |
|  | *SD* | | 1.11 | 1.11 |  |
| Tired eyes |  | |  |  |  |
|  | *M* | | 1.27 | 1.07 |  |
|  | *Md* | | 1.00 | 0 |  |
|  | *SD* | | 1.53 | 1.53 |  |
| Dizziness |  | |  |  |  |
|  | *M* | | 1.00 | .80 |  |
|  | *Md* | | 0 | 0 |  |
|  | *SD* | | 1.46 | 1.47 |  |
| Nausea |  | |  |  |  |
|  | *M* | | .40 | .40 |  |
|  | *Md* | | 0 | 0 |  |
|  | *SD* | | .91 | .74 |  |
| Difficulty focusing | | | |  |  |
|  | *M* | | .93 | .80 |  |
|  | *Md* | | 0 | 0 |  |
|  | *SD* | | 1.33 | 1.47 |  |
| Increased salivation | | |  |  |  |
|  | *M* | | .07 | 0 |  |
|  | *Md* | | 0 | 0 |  |
|  | *SD* | | .26 | 0 |  |
| *Notes*. *M* = mean value. *Md* = median. *SD* = standard deviation. Overall score can range from 0 to 42. Items can range from 0 to 6. Higher values indicate greater cyber sickness. | | | | |  |
|  | | | | |  |
